# Vpu serine 52 dependent counteraction of tetherin is required for HIV-1 replication in macrophages, but not in ex vivo human lymphoid tissue

**DOI:** 10.1186/1742-4690-7-1

**Published:** 2010-01-15

**Authors:** Michael Schindler, Devi Rajan, Carina Banning, Peter Wimmer, Herwig Koppensteiner, Alicja Iwanski, Anke Specht, Daniel Sauter, Thomas Dobner, Frank Kirchhoff

**Affiliations:** 1 Heinrich-Pette-Institute for Experimental Virology and Immunology, Martinistrasse 52, 20251 Hamburg, Germany; 2 Institute of Virology, University of Ulm, Albert-Einstein-Allee 11, 89081 Ulm, Germany; 3 Current address: Emory University, Atlanta GA 30322, USA

## Abstract

**Background:**

The human immunodeficiency virus type 1 (HIV-1) Vpu protein degrades CD4 and counteracts a restriction factor termed tetherin (CD317; Bst-2) to enhance virion release. It has been suggested that both functions can be genetically separated by mutation of a serine residue at position 52. However, recent data suggest that the S52 phosphorylation site is also important for the ability of Vpu to counteract tetherin. To clarify this issue, we performed a comprehensive analysis of HIV-1 with a mutated casein kinase-II phosphorylation site in Vpu in various cell lines, primary blood lymphocytes (PBL), monocyte-derived macrophages (MDM) and *ex vivo *human lymphoid tissue (HLT).

**Results:**

We show that mutation of serine 52 to alanine (S52A) entirely disrupts Vpu-mediated degradation of CD4 and strongly impairs its ability to antagonize tetherin. Furthermore, casein-kinase II inhibitors blocked the ability of Vpu to degrade tetherin. Overall, Vpu S52A could only overcome low levels of tetherin, and its activity decreased in a manner dependent on the amount of transiently or endogenously expressed tetherin. As a consequence, the S52A Vpu mutant virus was unable to replicate in macrophages, which express high levels of this restriction factor. In contrast, HIV-1 Vpu S52A caused CD4+ T-cell depletion and spread efficiently in *ex vivo *human lymphoid tissue and PBL, most likely because these cells express comparably low levels of tetherin.

**Conclusion:**

Our data explain why the effect of the S52A mutation in Vpu on virus release is cell-type dependent and suggest that a reduced ability of Vpu to counteract tetherin impairs HIV-1 replication in macrophages, but not in tissue CD4+ T cells.

## Background

Vpu is an accessory HIV-1 protein of 16-kDa expressed late during the viral life cycle [1], and it is known to perform two major functions. Firstly, Vpu targets CD4 for degradation in the endoplasmic reticulum [2-4]. Secondly, it promotes virion release in a cell-type dependent manner by counteracting a host restriction factor that can be induced by interferon-alpha [5]. This factor has been identified as CD317/BST-2 and is termed tetherin, because it "tethers" nascent virions to cell membranes [6,7]. From a mechanistic point of view Vpu binds to CD4, is phosphorylated at two serine residues at positions 52 and 56 by casein kinase II (CK-II), and recruits the E3-ubiquitin ligase substrate recognition factor β-TrCP. Subsequently, CD4 is ubiquitinated and degraded by the cellular proteasome [1,4,8]. Recent studies suggest that Vpu may induce internalization and degradation of tetherin by the same pathway [9-11]. In contrast, earlier work suggested that phosphorylation of S52 and S56 in the cytosolic domain of Vpu by CK-II is critical for CD4 degradation, but not for the enhancement of virion release [8,12-15]. Since the enhancing effect of Vpu on HIV-1 release is cell type dependent [5,16,17], some of these seeming discrepancies may result from different levels of tetherin expression and hence a differential requirement for effective tetherin antagonism.

In the present study, we performed a comprehensive analysis of Vpu function in HIV-1 infected primary cells and *ex vivo *tissue. In comparison to wildtype Vpu, the S52A mutant was strongly impaired in its ability to counteract tetherin, permitting viral release only at low levels of tetherin expression. These results may explain why HIV-1 encoding S52A Vpu caused CD4+ T-cell depletion and replicated with wildtype-like efficiency in lymphoid cells and HLT *ex vivo*, but not in macrophages that express higher levels of tetherin. In sum, our data suggest that the ability of Vpu to counteract tetherin is an important determinant for HIV-1 cell tropism.

## Results

### Vpu S52A impairs tetherin and CD4 degradation in transfected 293T cells

**Figure 1:**
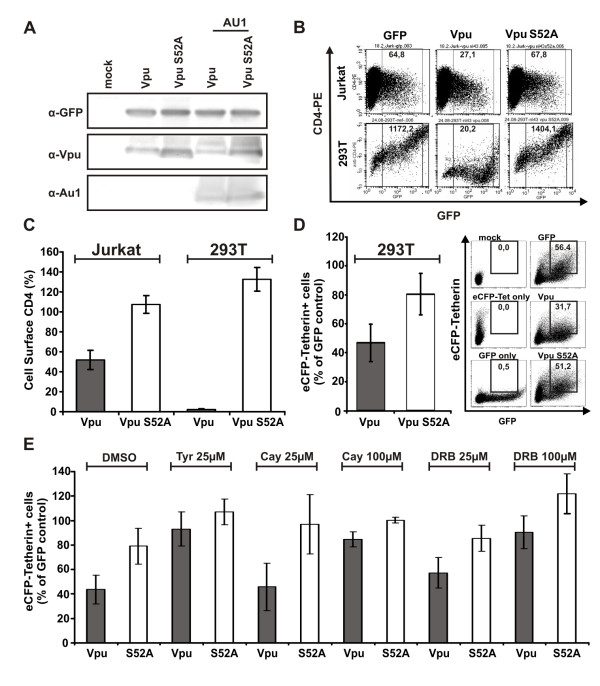
**Mutation of S52A impairs Vpu-mediated degradation of CD4 and tetherin**. (**A**) Western blot analysis of Vpu expression in lysates of transfected 293T cells. (**B**) FACS analysis of CD4 expression by Jurkat (upper panel) and CD4 co-transfected 293T cells (lower panel) expressing GFP alone or together with the Vpu and Vpu S52A proteins. Numbers give the MFI of the specified region (**C**) Quantitative analysis of CD4 downmodulation in Jurkat and 293T cells. Shown are the mean percentages of CD4 down-modulation +/- SD from six (Jurkat) and three (293T) independent experiments. Cell surface CD4 is given as a percentage of that measured on cells transfected with the control vector expressing GFP only (100%). (**D**) Quantitative analysis of tetherin degradation in 293T cells. Numbers give percentages of GFP+/eCFP+ cells in the specified region. Shown are the mean percentages of tetherin degradation from eight independent transfections. Values give percentages of cells co-expressing GFP and eCFP-tetherin. The mean values obtained with the GFP only control are set as 100%. (**E**) The same experimental setup as presented in **D**, however with different concentrations of the indicated CK-II inhibitors added during media change following transfection. Means and standard deviations are calculated from three to six independent transfection experiments.

For functional analyses, we generated untagged and AU1-tagged forms of the wildtype and S52A HIV-1 NL4-3 Vpus and verified their expression by Western blot analysis (Fig. 1A). Down-modulation of CD4 from the cell surface was measured by flow cytometric analysis of Jurkat T cells transiently transfected with vectors co-expressing Vpu and GFP via an internal ribosomal entry site (IRES). Transport of CD4 to the cell surface was measured by co-transfection of 293T cells with CD4 and constructs expressing GFP alone or together with Vpu. Wildtype Vpu caused about 2-fold reduced levels of CD4 expression on Jurkat T cells and efficiently blocked the transport of newly synthesized CD4 to the surface of 293T cells (Fig. 1B, C). In contrast, the S52A Vpu was inactive in both assays (Fig. 1B, C).

It has been shown that Vpu reduces the total levels of cellular tetherin, and it has been suggested that this effect may be important for its capability to promote virus release [9,10,18,19]. To test whether the S52A change affects tetherin degradation by Vpu, we generated an N-terminally eCFP-tagged version of tetherin. Confocal microscopy showed that the fusion protein had a subcellular localization comparable to endogenous tetherin and inhibited viral particle release (data not shown). Degradation of total cellular tetherin was measured by co-transfection of eCFP-tetherin with the various Vpu/GFP constructs. Expression of wildtype Vpu resulted in about 50% reduction in the number of tetherin expressing cells, whereas the S52A Vpu degraded tetherin in only about 20% of cells (Fig. 1D). It has been shown that Vpu is phosphorylated by CK-II [12], but the importance of an active CK-II for the ability of Vpu to degrade tetherin is not known. Therefore, we measured Vpu-mediated tetherin degradation in the presence of different CK-II inhibitors (Fig. 1E). Tyrphostin inhibited degradation of tetherin by Vpu already at 25 μM whereas Cay10577 and DRB did so in a dose-dependent manner, demonstrating the importance of CK-II activity for the degrading effects of Vpu on tetherin (Fig. 1E). These results show that mutation of S52A is sufficient to entirely disrupt the effect of Vpu on CD4 and establish at a single cell level that an intact CK-II phosphorylation site as well as active CK-II are important for degradation of tetherin by Vpu.

### The S52A Vpu is only able to antagonize tetherin at low expression levels

**Figure 2:**
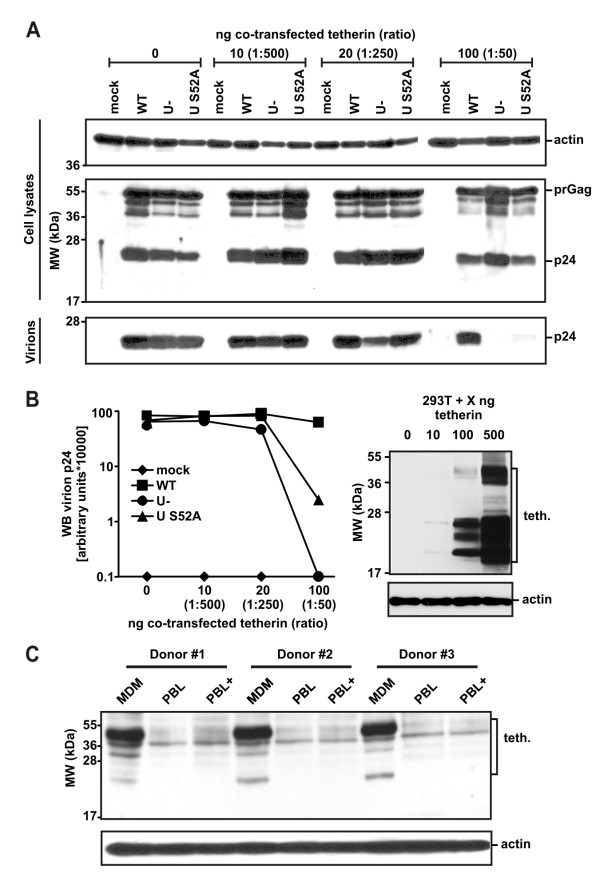
**Vpu S52A dose-dependently counteracts tetherin in transfected 293T cells**. (**A**) WB analysis of cellular lysates transfected with the indicated HIV-1 proviral constructs and different concentrations of tetherin plasmid. Viral supernatants were harvested two days post transfection, filtered and pelleted. Lysed cells and virus stocks were blotted for the presence of p24 and actin as a loading control. (**B**) Quantification of p24 release by the proviral constructs in the presence of different amounts of tetherin and analysis of tetherin transfected 293T cells. Presented is one out of two independent WB experiments showing the same results. Abbreviations, U-, Vpu-defective; S52A, VpuS52A. (**C**) Western blot analysis of endogenous tetherin expression in PBL and MDM from three different donors. PBL were either left untreated or stimulated with 1 μg/ml PHA for 24 hours (PBL+).

Vpu S52A still degraded tetherin to some extent in cells co-transfected with Vpu and tetherin expression plasmids (Figures 1D and 1E). Therefore, we speculated that Vpu S52A might be able to enhance HIV-1 release at low levels of tetherin expression. We co-transfected 293T cells with WT, Vpu-defective, and Vpu S52A expressing proviral constructs and different amounts of tetherin ranging from 100 ng (1:50; ratio transfected tetherin:provirus) to 10 ng (1:500); and we measured cellular as well as released p24 by a quantitative Western blot two days later (Fig. 2A and 2B). As expected, 293T cells expressing very low 10 ng (1:500) levels of tetherin released p24 independently of functional Vpu expression. However, transfection of 20 ng (1:250) tetherin already reduced virus release of Vpu-defective HIV-1 by about 50%. At these levels of tetherin expression the S52A Vpu enhanced p24 release as efficiently as the wildtype Vpu protein. In contrast, virus release of the mutant was suppressed by more than one order of magnitude at higher levels of tetherin expression (Fig. 2A, B). Of note, we did not detect any p24 in the supernatant of cells expressing Vpu-defective HIV-1 when tetherin was transfected at a ratio of 1:50. As a control, we measured virion content by ELISA in supernatants of transfected cells before the virus was pelleted. These analyses demonstrated that results obtained by ELISA correlated highly significantly (R = 0.9159; p < 0.0001) with the quantitative WB results (Additional file 1). In sum, the S52A change severely attenuates the ability of Vpu to enhance HIV-1 release with increasing levels of tetherin expression.

Previously, it was reported that macrophages and primary T-cells, the main HIV-1 target cells *in vivo*, express different amounts of endogenous tetherin [20]. Prompted by our results, we speculated that Vpu with a mutated CK-II site might not be able to counteract high levels of tetherin expression found in macrophages, but may replicate efficiently in T-cells that express low levels of tetherin. Since it is known that macrophages exert phenotypically high donor variations, we first aimed to investigate the levels of endogenous tetherin in macrophages from various donors in comparison to autologous T-cells (Fig. 2C). Western blot analysis revealed multiple bands, which is in agreement with previous findings showing that tetherin is glycosylated and can multimerize [10,18,20]. Untransfected 293T cells that allow efficient release of HIV-1 particles in the presence and absence of Vpu did not express detectable levels of tetherin (Fig. 2B). Of note, macrophages expressed markedly higher levels of tetherin than PHA-stimulated or unstimulated PBL (Fig. 2C). Thus, Vpu S52A might be differentially active in the enhancement of particle release from primary T-cells and monocyte-derived macrophages (MDM) because it is only able to counteract tetherin at low expression levels.

### Vpu S52A promotes virus release from HeLa-derived cells

**Figure 3:**
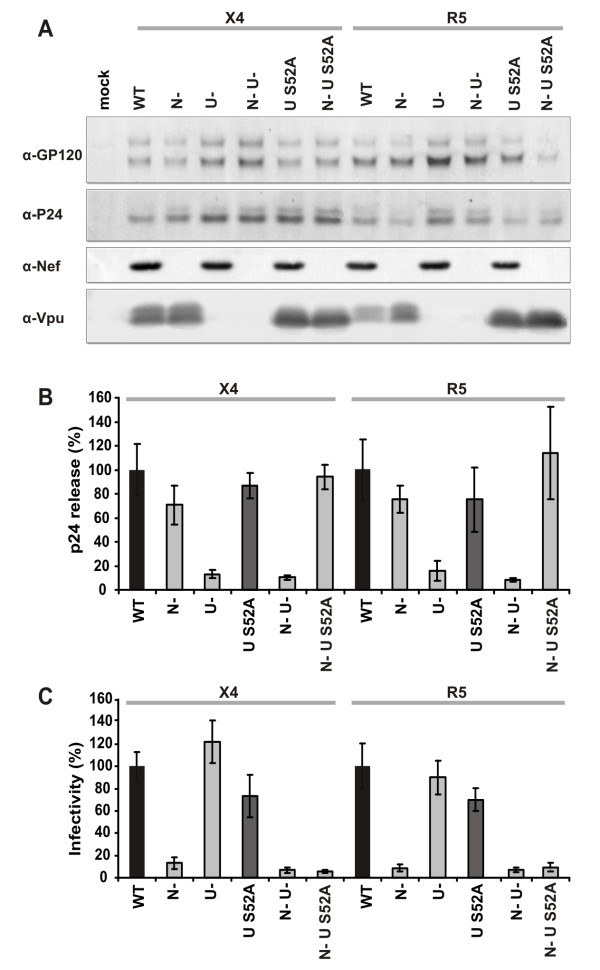
**Vpu S52A does not impair HIV-1 release from P4-CCR5 cells**. (**A**) Western blot analysis of viral gene expression in lysates of transfected 293T cells. (**B**) Viral particle release by P4-CCR5 cells transfected with the indicated X4 and R5 HIV-1 NL4-3 proviral constructs. P4-CCR5 cells were transfected with 0.1 μg proviral DNA in sextuplicates and p24 in the culture supernatants was quantified by p24 ELISA three days later. Measurement of the β-Gal activities in the cell lysates verified similar transfection efficiencies (not shown). Values give averages +/- SD from two independent experiments with sextuplicate transfections and represent percentages compared to NL4-3 wildtype transfected cells (100%). (**C**) P4-CCR5 indicator cells were infected in triplicate with virus stocks containing 1 ng p24 antigen derived from 293T cells transfected with the indicated proviral constructs and β-Gal activity was determined three days later. Shown are average values +/- SD from two independent experiments with triplicate infections of two independent virus stocks. Infectivity is given as percentage compared to infectivity of NL4-3 wildtype infected cells (100%). Abbreviations, N-, Nef-defective; U-, Vpu-defective; S52A, VpuS52A.

To investigate the effect of the S52A mutation in Vpu on HIV-1 release we constructed CXCR4(X4)- and CCR5(R5)-tropic HIV-1 NL4-3 mutants carrying this change alone or in combination with a disrupted *nef *gene. The latter constructs were generated because Nef is known to down-modulate CD4 and to enhance viral infectivity and replication and may thus bias possible effects of the S52A change in Vpu [21-23]. Western blot analyses confirmed that all proviral constructs showed the expected differences in Vpu and Nef expression (Fig. 3A). Next, we decided to assess first the release of the different HIV-1 NL4-3 variants in the well established HeLa-derived P4-CCR5 cells [24,25]. We transfected them with normalized quantities of proviral DNA and measured p24 content in the cell culture supernatant. Importantly, transfection efficiencies were comparable, since similar levels of Tat-dependent expression of the LTR-driven β-galactosidase gene were detected in all cell lysates (data not shown). In agreement with the previous finding that Vpu is required for effective virus release from HeLa-derived cell lines [16], the expression of wildtype Vpu resulted in about 5- to 6-fold increased levels of p24 antigen in the culture supernatant. The S52A Vpu enhanced the release of progeny virions with similar efficiency, whereas Nef had no significant effect (Fig. 3B). This result was in line with our hypothesis that Vpu S52A can overcome relatively low levels of tetherin expression, because our P4-CCR5 cells expressed tetherin in a range comparably to unstimulated PBMCs (Additional file 2). Infection of P4-CCR5 cells with virus stocks containing normalized amounts of p24 (1 ng p24) [25] showed that only changes in *nef*, but not in *vpu*, impaired viral infectivity (Fig. 3C). Most importantly, these findings demonstrated that the S52A Vpu is capable of enhancing virion release from HeLa derived P4-CCR5 cells that express relatively low levels of tetherin.

### The S52A mutation in Vpu does not impair HIV-1 replication and cytopathicity in lymphoid tissue *ex vivo*

**Figure 4:**
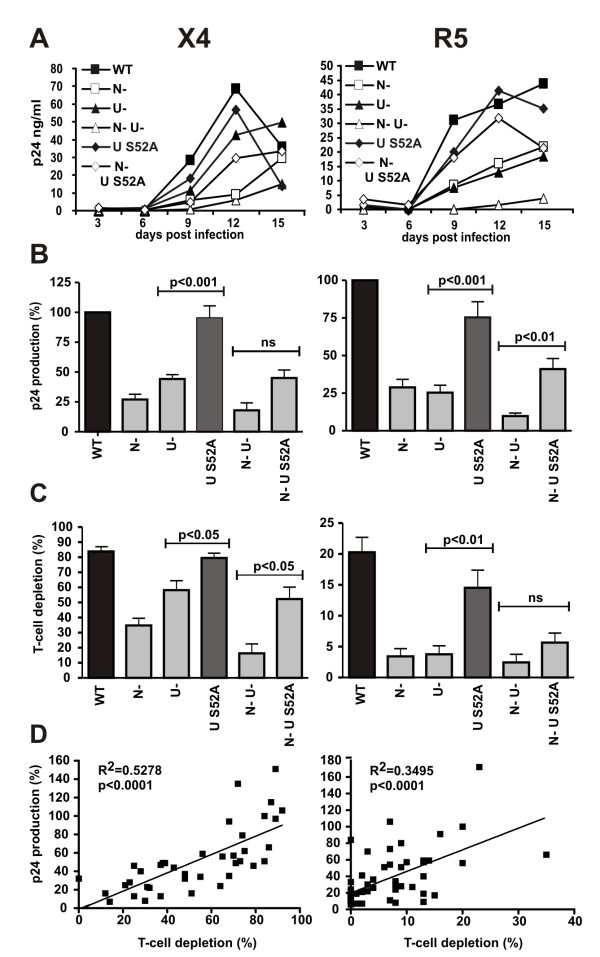
**Vpu S52A is dispensable for HIV-1 replication and cytopathicity in *ex vivo *infected HLT**. Representative replication kinetics (**A**) of the indicated X4 and R5 HIV-1 NL4-3 constructs. (**B**) Cumulative p24 production over 15 days and (**C**) CD4+ T cells depletion at the end of culture in tissues from eight (X4) and ten (R5) donors infected with the indicated HIV-1 variants. Values are given as percentages compared to cultures infected with NL4-3 wildtype (100%). Shown are means +/- SEM. (**D**) Correlation between p24 production and CD4+ T-cell depletion.

It has been demonstrated that Vpu is critical for efficient HIV-1 replication and CD4+ T-cell depletion in HLT *ex vivo *[26,27]. This system allows productive HIV-1 infection without exogenous stimulation and mimics infection of lymphatic tissues, one of the major sites of viral replication *in vivo *[28]. To study the effect of the S52A change in Vpu on HIV-1 replication and cytopathicity, we infected HLT *ex vivo *with the X4 and R5 NL4-3 variants (Fig. 3A). Representative examples of replication results are presented in Figure 4A. Overall, we found that a defective *vpu *gene reduced the production of wildtype X4 NL4-3 by 60% and of the R5-tropic derivative by 75% (Fig. 4B). Similarly, deletion of *nef *reduced cumulative virus production by about 75% (Fig. 4B). In contrast, the HIV-1 S52A Vpu mutation did not significantly attenuate HIV-1 replication (Fig. 4A and 4B). Consistent with the results of previous studies [26,27], wildtype X4 NL4-3 virus depleted the *ex vivo *infected tissues of 80% of X4-expressing CD4+ T cells, whereas the R5 HIV-1 derivative depleted 20% of R5+/CD4+ cells (Fig. 4C). Individual or combined deletions in *nef *and *vpu *significantly reduced CD4+ T-cell depletion irrespectively of the viral coreceptor tropism, whereas the S52A mutation in Vpu had no significant effect (Fig. 4C). The efficiency of viral replication correlated well with CD4+ T-cell depletion (Fig. 4D) suggesting that these differences in cytopathicity resulted from lower numbers of infected cells, rather than from direct effects of Nef or Vpu on cell killing. These data show for the first time that the CK-II phosphorylation site in Vpu is not critical for effective viral spread and CD4+ T-cell depletion in *ex vivo *infected lymphoid tissue.

It has previously been established that HIV-1 replication in HLT occurs mainly in both activated and non-activated CD4+ T-cells [29] that express relatively low levels of tetherin (Fig. 2C, Additional file 2). Therefore, the wildtype like phenotype of HIV-1 Vpu S52A in HLT might be due to low tetherin expression levels in the relevant HIV-1 tissue target cells. Since it is difficult to isolate a sufficient number of CD4+ T-cells from these tissues to directly assess endogenous tetherin levels, we decided to investigate if replication of the HIV-1 variants in PBL mimics the situation in HLT. As expected, HIV-1 Vpu S52A replicated as efficiently as WT HIV-1 in cultures of primary blood lymphocytes, whereas Vpu-defective HIV-1 showed attenuated and delayed replication kinetics (Additional file 3 fig. S3a). Furthermore, electroporation of Jurkat T-cells with the proviral constructs and increasing amounts of tetherin expression plasmids confirmed that in T-cells the ability of Vpu S52A to enhance HIV-1 release also decreases in a tetherin-expression dependent manner (Additional file 3 fig. S3B).

### The S52A change in Vpu impairs HIV-1 replication in macrophages

**Figure 5:**
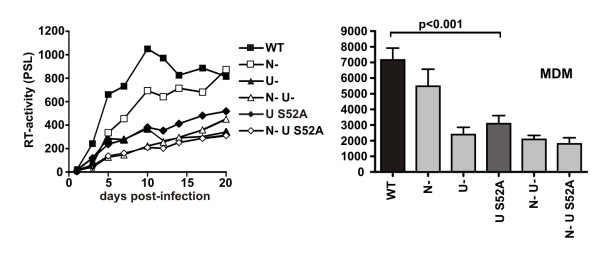
**Vpu S52A impairs HIV-1 replication in macrophages**. Replication kinetics of wildtype NL4-3 and the indicated mutants in monocyte-derived macrophages and average levels of cumulative RT production by macrophages infected with the NL4-3 variants over a 20 day period. Values give averages +/- SEM of macrophages from three different donors with two independent virus stocks containing 1 ng p24 antigen. PSL, photon-stimulated luminescence.

Macrophages express markedly higher levels of tetherin than PHA-stimulated or unstimulated PBL (Fig. 2C, Additional file 2). Thus, we finally wanted to challenge the hypothesis that Vpu S52A might be impaired in the enhancement of particle release from infected MDM, because it is not able to counteract high tetherin expression levels. Therefore, we investigated the replicative capacity of the different R5-tropic viruses (Fig. 3A) in MDMs. In agreement with previous reports [30-33], only the disruption of *vpu *but not of *nef *severely attenuated HIV-1 replication (Fig. 5). Most remarkably, the S52A mutation in Vpu impaired the replicative capacity of HIV-1 in macrophages as severely as the complete lack of Vpu function. Thus, Vpu S52A might be impaired in the enhancement of particle release from infected MDM, because it is not able to counteract tetherin at high expression levels.

### Modulation of cell surface expressed CD4 and tetherin in HIV-1 infected PBL and macrophages

**Figure 6:**
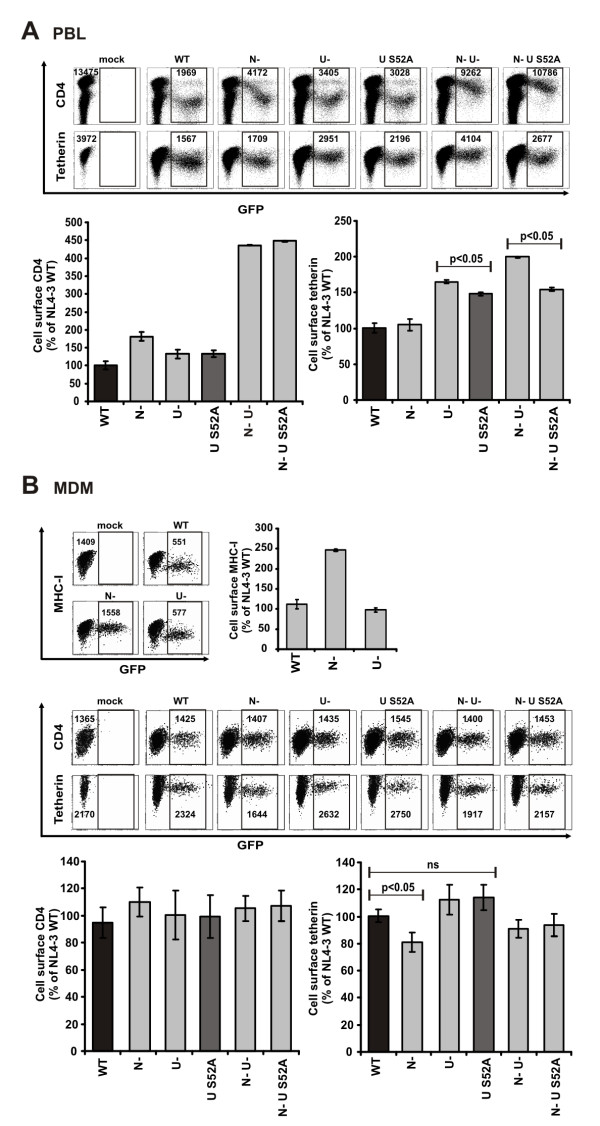
**Modulation of tetherin and CD4 in primary T-cells and macrophages by Vpu**. (**A**) FACS analysis of CD4 and tetherin modulation in infected PBL cultures. PBL were infected with HIV-1 variants expressing eGFP via an IRES. Cells were stained with antibodies and measured by flow cytometry three days later. To quantify modulation of cell surface expressed CD4 and tetherin MFI of PBLs infected with HIV-1 NL4-3 WT was set as 100%. Depicted are means +/- SD derived from experiments with four different donors. (**B**) Primary macrophages were infected with the indicated R5-tropic virus stocks expressing eGFP via an IRES. Cells were analyzed for cell surface MHC-I, CD4 and tetherin five days post infection similar to the PBL cultures. Presented are means +/- SD from infections with macrophages from three different donors each of those were infected with two independent virus stocks.

Currently, it is not known whether Vpu modulates cell surface expression of tetherin in primary T-cells and macrophages. To address this, we generated proviral HIV-1 constructs containing wildtype or mutated *vpu *genes co-expressing Nef and eGFP via an IRES [25,34]. PBL and MDM were infected with VSV-G pseudotyped viruses and assessed for the modulation of cell surface CD4 and tetherin by FACS. In agreement with previous reports [21,22], we found that inactivation of Nef more severely reduced than Vpu the ability of HIV-1 to remove CD4 from the surface of infected primary T-cells (Fig. 6A). Nevertheless, the fact that the combined deletions had the most disruptive effects on cell surface CD4 expression demonstrated that both Nef as well as Vpu are important for effective removal of CD4. Moreover, the S52A change as well as inactivation of Vpu impaired the ability of HIV-1 to down-modulate CD4 to the same extent (Fig. 6A, left). Down-modulation of cell surface tetherin from HIV-1 infected PBL was clearly dependent on Vpu expression (Fig. 6A, right). Furthermore, the levels of cell surface tetherin in infected cells expressing S52A Vpu were significantly lower than in cells infected with HIV-1 containing an entirely defective *vpu *gene (Fig. 6A, right). Thus, Vpu S52A down-modulates tetherin from HIV-1 infected T-cells, albeit with lower efficiency than wildtype Vpu.

Next, we assessed if our viruses allow us to investigate the modulation of cell surface expressed receptors in macrophages, and we measured the down-modulation of MHC-I as a control. Inactivation of Nef resulted in about 2.5 fold higher MHC-I surface levels compared to WT infected MDM (Fig. 6B). Surprisingly, CD4 expression levels in HIV-1 infected MDM were comparably to uninfected cells, irrespective of Vpu or Nef expression (Fig 6B, left). Moreover, Vpu as well as the S52A mutant had similar minor effects on the levels of cell surface tetherin in MDM (Fig. 6B, right). Notably, MDMs infected with Nef-defective HIV-1 expressed lower levels of tetherin (Fig. 6B, right). Thus, Nef seems to induce tetherin cell surface expression in HIV-1 infected macrophages, perhaps as a result of Nef induced release of inflammatory cytokines [35].

In summary, our experiments demonstrate that VpuS52A reduces the levels of cell surface expressed tetherin in PBL, whereas it does not in macrophages.

## Discussion

In the present study we demonstrate that the S52A mutation in Vpu impairs the ability of HIV-1 to replicate in macrophages, but not in *ex vivo *infected HLT cultures or PBL. This difference is most likely due to a reduced capability in counteracting tetherin, as the S52A Vpu mutant virus showed a wildtype phenotype in cells that express relatively low levels of this restriction factor, i.e. P4-CCR5 and T-cells, and a *vpu*-defective phenotype in cells that express higher levels, such as macrophages, 293T and T-cells transiently transfected with relatively high amounts of tetherin expression plasmids.

These data explain why it has been controversial whether the CK-II phosphorylation site in Vpu is only critical for CD4 degradation or is also relevant for virion release [8,12-15]. Indeed, we and others have found that S52 in Vpu is involved in the down-modulation and the degradation of tetherin (Fig. 1, 6) [7,9-11,19]. While most groups investigated Vpu with mutations in both serines at positions 52 and 56 (S2/6), we utilized the Vpu S52A mutant in our experiments. In the 293T experiments, S52A showed a similar phenotype like S2/6 (Fig. 1A-D and data not shown), which is in agreement with a recent report that also utilized the S52A variant [11]. This suggests that mutation of S52 alone is sufficient to disrupt the CK-II phosphorylation site in Vpu. Furthermore, we establish that phosphorylation by CK-II is clearly important for Vpu to degrade tetherin by the use of three different CK-II inhibitors (Fig. 1E).

One possible explanation of the remaining anti-tetherin activity of the S52A mutant is that Vpu uses alternative pathways to counteract the restriction factor. On the other hand, Vpu containing mutations at the serine residues at position 52 and 56 has been shown to be able to bind to tetherin [10]. This could explain why the S52A Vpu exerts some residual counteracting activity, despite the fact that it does not efficiently induce tetherin degradation.

More importantly, our data suggest that the ability of Vpu to counteract tetherin is particularly required for HIV-1 replication in macrophages which are involved in virus transmission, the establishment of viral reservoirs, and neurological disorders associated with HIV-1 infection [36-38]. Thus, a reduced capability of Vpu to antagonize tetherin and to promote the release of progeny virions from macrophages may have important consequences for HIV-1 transmission and pathogenicity. This is also highlighted by a recent report, demonstrating that only pandemic HIV-1 M expresses a fully functional Vpu protein, whereas the rarely distributed HIV-1 N and O groups contain Vpu proteins that either are impaired in CD4 or tetherin degradation [39]. Conversely, it is remarkable that HIV-1 expressing a Vpu protein which is severely impaired in its ability to counteract tetherin, replicates efficiently in PBL and HLT and depletes CD4+ T-cells, particularly since Vpu is considered as a target for antiviral therapy [40]. Thus, Vpu inhibitors might need to be combined with agents inducing tetherin to achieve significant beneficial effects. *In vitro *this can be achieved by treatment of human cells with interferon-alpha [6,20]. Interestingly, interferon-alpha is upregulated by HIV-1 infection [41,42] which may subsequently lead to the induction of tetherin in a feedback mechanism. Indeed we observed strong attenuation of viral replication in HLT and PBL in the presence of 100 U/ml interferon-alpha, irrespective of an intact *vpu *gene (data not shown). This is in line with other reports [38-40] and could be explained by the fact that a variety of genes are upregulated in response to interferon-alpha, and additional pathways are triggered that might interfere with HIV-1 production [40-43].

Interestingly, among the predominant HIV-1 target cells *in vivo*, tetherin is highly expressed on macrophages (this study, [20]) and dendritic cells [43,44]. Thus, the ability of HIV-1 to efficiently counteract tetherin might have an impact on the cellular tropism of the virus. Both cell types become HIV-1 infected by the usage of the CCR5 co-receptor. Thus, it is also tempting to speculate that viral co-receptor tropism, i.e. the usage of CCR5 for viral entry segregates with the ability of Vpu to efficiently counteract tetherin. As already mentioned above, tetherin might be induced during HIV-1 infection by interferon-alpha, whose serum levels correlate with disease progression [45-47]. Therefore, our data carefully raise the possibility that the emergence of CXCR4 using HIV-1 variants during infection [48], might at least in part be also driven by increased expression of tetherin on the target cells. Currently it is not known whether primary HIV-1 *vpu *alleles differ in their ability to counteract tetherin. To challenge these hypotheses, studies investigating the anti-tetherin activity of HIV-1 *vpu *alleles from viruses isolated during different stages of infection and with different co-receptor tropism are warranted.

## Methods

### Plasmids and proviral constructs

For functional analysis, we generated vectors co-expressing Vpu or VpuS52A and GFP from a single bicistronic RNA via an internal ribosome entry site (IRES), as initially described for the analysis of Nef function [49]. Briefly HIV-1 NL4-3 Vpu was amplified with primers introducing unique XbaI and MluI restriction sites and subcloned into the pCGCG-IRES-GFP vector [50]. AU1-tagged Vpu and VpuS52A variants were constructed by introducing the DTYRYI-sequence at the C-terminus together with the MluI primer. Site directed mutagenesis was utilized to introduce the S52A change in NL4-3 Vpu. The HIV-1 NL4-3 proviral constructs carrying disrupting mutations in *nef, vpu *or both viral genes have been previously described [27]. Splice overlap extension PCR was used to introduce mutation S52A in HIV-1 NL4-3 *vpu *and the element was subcloned by using the unique restriction sites StuI in *env *and the PflmI site just downstream of the *pol *gene, respectively. R5-tropic HIV-1 NL4-3 variants were constructed by exchanging the V3-loop region of NL4-3 with the one from the R5-tropic 92th014.12 isolate [51] by using the unique restriction sites StuI and NheI. HIV-1 NL4-3 variants co-expressing eGFP via an IRES were constructed by subcloning of fragments containing mutations in *nef *or *vpu *in the pBR-NL4-3-IRES eGFP backbone [25,34]. The pECFP-tetherin construct was cloned by amplification of tetherin from a cDNA library (Spring Bioscience) introducing the single cutter restriction sites *XhoI *and *EcoRI*. The fragment was cloned in the pECFP-C1 vector backbone (Clontech). An untagged tetherin plasmid was cloned by amplification of tetherin with primers introducing *XbaI *and *MluI *sites and subcloning in the pCGCG vector [50]. The IRES-GFP cassette was removed by digestion and religation with *BamHI*. The integrity of all PCR-derived inserts was verified by sequence analysis.

### Cell culture, transfections, virus stocks, p24 release and infectivity assays

P4-CCR5, 293T and Jurkat cells were cultured as described previously [25,50]. P4-CCR5 and 293T cells were maintained in Dulbecco's modified Eagle's medium containing 10% heat-inactivated fetal bovine serum. The human Jurkat T-cell line was cultured in RPMI1640 medium supplemented with 10% fetal calf serum and antibiotics. PBMC were generated by Ficoll gradient centrifugation [34] and PBLs were recovered post plastic adherence of monocytes. To generate primary macrophage cultures PBMCs from healthy human donors were isolated using lymphocyte separation medium and macrophages were generated in teflon tubes (CellGenix) and cultured as described before [52,53]. Transfection of Jurkat T-cells was performed using the DMRIE-C reagent (Invitrogen, Gibco) following manufacturer's instructions. Furthermore, electroporation of Jurkat T-cells with proviral constructs and tetherin expression plasmids was performed with the MP-100 microporator device (PeqLab) as recommended by the manufacturer. Briefly, 4 μg of proviral constructs co-expressing GFP were electroporated with the indicated amounts of tetherin plasmid. Two days post infection GFP+ cells were determined by FACS and the amount of released p24 was quantified in the supernatants using a p24 ELISA provided by the "AIDS & Cancer virus program" (NCI, Frederick). P4-CCR5-cells were transfected using magnetic assisted transfection (IBA Tagnology) following standard protocols of the manufacturer. Briefly, 4000 P4-CCR5 cells per well were sown into 96-well plates one day prior to transfection. For transfection 0.1 μg of proviral DNA was co-incubated with 0.1 μl MaTRA-A reagent in 15 μl OMEM (optimized minimum essential media, GIBCO) per well for 30 min. Three days post transfection supernatants were harvested and analyzed for p24 antigen concentrations and β-galactosidase activity. To generate viral stocks, 293T cells were transfected with the proviral NL4-3 constructs by the calcium chloride method as already described [25,34]. Virus stocks and supernatants of transfected or infected cells to assess p24 release were quantified using the p24 ELISA described above. Virus infectivity was determined using P4-CCR5 cells as described [25]. Briefly, 4000 cells per well were sown out in 96-well-dishes in a volume of 100 μl and infected after overnight incubation with virus stocks containing 1 ng of p24 antigen. Three days post-infection viral infectivity was detected using the Gal screen kit from TROPIX as recommended by the manufacturer. β-galactosidase activities were detected as relative light units per second (RLU/s) in a microplate reader.

### Flow cytometric analysis

CD4 and GFP reporter expression levels in Jurkat cells co-expressing Vpu and eGFP were measured as described previously for the analysis of Nef function [50]. Retention of newly synthesized CD4 from the endoplasmic reticulum to the cell surface in 293T cells was measured by standard calcium chloride co-transfection of 1 μg pCDNA-CD4 plasmid with 4 μg pCG plasmid expressing Vpu, VpuS52A or GFP only. Cells were harvested and stained for FACS analysis 2 days post transfection essentially as described previously [50]. pECFP-tetherin and GFP expression in 293T cells were analyzed similar to CD4 expression, but on a FACSAria equipped with a 405 nm laser. For the CK-II inhibition experiments, we used Tyrphostin AG1112 (Sigma), Cay10577 (Biozol) and DRB (EnzoLife) reconstituted in DMSO. The concentrations used did not induce cytotoxic effects as determined by FACS FSC/SSC and MTT test (data not shown). PBLs and MDMs were infected with VSVG pseudotyped virus stocks containing 50 ng p24. PBLs were analyzed by flow cytometry three days post infection for CD4 and tetherin expression as already described [34]. Similarly, primary macrophage cultures were trypsinized five days post infection and stained for CD4 and MHC-I expression as before [54]. Cell surface tetherin was measured by staining of PBLs or MDMs with 1:50 dilutions of the anti-tetherin/CD317 HM1.24 mAb kindly provided by Chugai Pharmaceuticals. As secondary antibody we used an 1:100 diluted Alexa633-conjugated goat anti-mouse antibody (Invitrogen).

### Immunoblotting

293T cells were transfected with 5 μg of vector DNA as described above. Two days post-transfection cells were pelleted, lysates were generated and separated through 12% SDS-PAGE. Expression of Vpu in whole cellular lysates was analyzed by immunoblot using 1:500 diluted mouse anti-AU1 AB (Covance) or 1:500 dilutions of rabbit anti-Vpu sera kindly provided by U.Schubert [55]. GFP was detected with a 1:2000 dilution of rabbit anti-GFP AB (Abcam). Viral envelope, GAG and Nef was visualized by 1:5000 dilutions of human-anti-HIV-1 gp120 Ab2G12, 1:5000 diluted rabbit-anti-HIV-1 p24 (provided by the AIDS & Cancer virus program) and 1:1000 dilutions of mouse-anti-HIV-1 Nef (aa151-170) (ABI). For WB-analyses of cellular tetherin we lysed 293T cells that were transfected with different amounts of tetherin as well as P4-CCR5, PBMC, PBL and MDMs and quantified total protein content using the 2D-quant kit (Amersham Biosciences). 40 μg protein of each lysate were separated on a 15%-SDS-bisacrylamide gel. For analysis of tetherin we used mouse anti-tetherin (B01P, Abnova) at a concentration of 1:500 and secondary goat anti-mouse (Jackson Immuno Research) at 1:10000 for ECL detection. To analyze enhancement of p24 release in the presence of tetherin we transfected 400.000 293T cells with proviral constructs (5 μg) and different amounts of tetherin (10, 20 and 100 ng). Two days post infection, we pelleted the filtered supernatants and lysed them as well as the producer cells with 50-100 μl RIPA buffer. Western blots were performed as described above, and band intensity was quantified using the Gene-snap software.

### *Ex vivo*-infected HLT

HIV-1 replication and cytopathicity in *ex vivo-*infected HLTwas determined as described previously [26,27]. Briefly, human tonsillar tissue removed during routine tonsillectomy was received within 5 hours of excision. The tonsils were washed thoroughly with medium containing antibiotics and sectioned into 2- to 3-mm^3 ^blocks. These tissue blocks were placed on top of collagen sponge gels and infected with virus stocks containing 0.5 ng p24 antigen essentially as described previously [26,27]. Supernatants were collected at three day intervals and productive HIV-1 infection was assessed by measuring p24 antigen content. Flow cytometry was performed on cells mechanically isolated from control and infected tissue blocks and depletion of CD4+ T-cells was quantified as described previously [26,27]. For determination of the CD4+/CD8+-T-cell ratio, cells were stained for surface markers by using anti-CD3 fluorescein isothiocyanate (FITC), anti-CD4-allophycocyanin (APC), and anti-CD8 Tri color.

### Viral replication in primary macrophage and PBL cultures

Human monocyte-derived macrophages (MDM) were isolated as described in "cell culture, virus stocks and transfection" and infected with 1 ng p24 of R5-tropic HIV-1 NL4-3 variants. To assess viral spread and replication, aliquots of the infected cell culture supernatants were taken in two to three day intervals and stored at -20°C. Viral replication was determined by RT-assay essentially as described previously [25]. Similarly, PBLs were infected with 1 ng p24 of X4-tropic HIV-1 NL4-3 variants expressing GFP via an IRES. For measurement of viral spread and transmission we took aliquots of infected cells in two or three days intervals and determined the amount of GFP+ cells by FACS as before [25].

### Statistical analysis

All statistical calculations were performed with a one-way analysis of variances (ANOVA) using Graph Pad Prism Version 5.0. Correlations were calculated with the linear regression module.

## Competing interests

The authors declare that they have no competing interests.

## Authors' contributions

Conceived and designed the experiments: MS FK. Performed the experiments: MS DR CB PW HK AI AS DS. Analyzed the data: MS DR CB PW FK. Contributed reagents/materials/analysis tools: MS TD FK. Wrote the paper: MS.

## Supplementary Material

Additional file 1 **Supplementary Figure S1. Assessment of viral release by quantitative WB correlates with p24 ELISA**. Correlation of the quantitative WB data shown in Fig. 2 with ELISA results, that were measured before the supernatants were pelleted. Click here for file

Additional file 2 **Supplementary Figure S2. HeLa derived P4-CCR5 cells express low levels of tetherin**. Western blot analysis of endogenous tetherin expression in primary cells and HeLa-derived P4-CCR5 cells. PBMC were either left untreated or stimulated with 1 μg/ml PHA for 24 hours (PBMC+). Click here for file

Additional file 3 **Supplementary Figure S3. Vpu S52A is dispensable for HIV-1 release in primary blood lymphocytes (PBL) and Jurkat T-cells**. (**A**) Replication kinetic of the indicated X4-tropic HIV-1 isolates expressing eGFP via an IRES in PBL cultures. PBLs were infected with 1 ng p24 and analyzed for the amount of GFP+ cells in two or three days intervals. Means +/- SD are calculated from infections of PBLs from two donors with three independent virus stocks. (**B**) The ability of Vpu S52A to enhance HIV-1 release from Jurkat T-cells is inhibited in a tetherin-dependent manner. 1*10^6 Jurkat cells were electroporated with the different proviral constructs coexpressing GFP and the indicated amount of tetherin expression plasmid as described in the methods section. Two days post electroporation the percentage of GFP+ cells as well as p24 contents of the supernatants were quantified. Presented are means and standard deviations from triplicate electroporations from one representative out of three independent experiments. Click here for file
