# Supplementary material for: Vpu serine 52 dependent counteraction of tetherin is required for HIV-1 replication in macrophages, but not in ex vivo human lymphoid tissue
Source: Retrovirology. 2010 Jan 15;7:1. doi: 10.1186/1742-4690-7-1 (PMC2823648; doi:10.1186/1742-4690-7-1)
Supplement: Additional file 1 — Supplementary Figure S1. Assessment of viral release by quantitative WB correlates with p24 ELISA. Correlation of the quantitative WB data shown in Fig. 2 with ELISA results, that were measured before the supernatants were pelleted. [file 1742-4690-7-1-S1.PDF]

Figure S1

Schindler et al

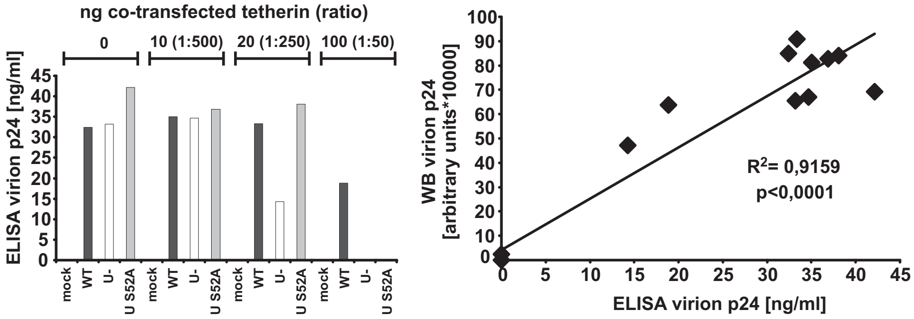

Supplementary Figure S1. Assessment of viral release by quantitative WB correlates with p24 ELISA. Correlation of the quantitative WB data shown in Fig. 6 with ELISA results, that were measured before the supernatants were pelleted.
