# Supplementary material for: Vpu serine 52 dependent counteraction of tetherin is required for HIV-1 replication in macrophages, but not in ex vivo human lymphoid tissue
Source: Retrovirology. 2010 Jan 15;7:1. doi: 10.1186/1742-4690-7-1 (PMC2823648; doi:10.1186/1742-4690-7-1)
Supplement: Additional file 2 — Supplementary Figure S2. HeLa derived P4-CCR5 cells express low levels of tetherin. Western blot analysis of endogenous tetherin expression in primary cells and HeLa-derived P4-CCR5 cells. PBMC were either left untreated or stimulated with 1 μg/ml PHA for 24 hours (PBMC+). [file 1742-4690-7-1-S2.PDF]

Figure S2

Schindler et al

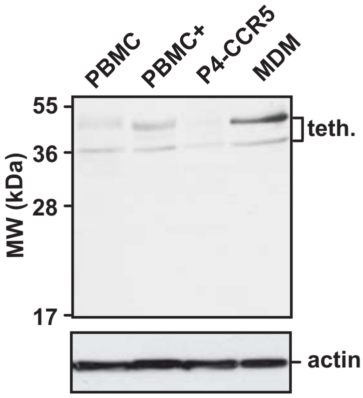

Supplementary Figure S2. HeLa derived P4-CCR5 cells express low levels of tetherin. Western blot analysis of endogenous tetherin expression in primary cells and HeLa-derived P4-CCR5 cells. PBMC were either left untreated or stimulated with 1μg/ml PHA for 24 hours (PBMC+).
