# Supplementary material for: Vpu serine 52 dependent counteraction of tetherin is required for HIV-1 replication in macrophages, but not in ex vivo human lymphoid tissue
Source: Retrovirology. 2010 Jan 15;7:1. doi: 10.1186/1742-4690-7-1 (PMC2823648; doi:10.1186/1742-4690-7-1)
Supplement: Additional file 3 — Supplementary Figure S3. Vpu S52A is dispensable for HIV-1 release in primary blood lymphocytes (PBL) and Jurkat T-cells. (A) Replication kinetic of the indicated X4-tropic HIV-1 isolates expressing eGFP via an IRES in PBL cultures. PBLs were infected with 1 ng p24 and analyzed for the amount of GFP+ cells in two or three days intervals. Means +/- SD are calculated from infections of PBLs from two donors with three independent virus stocks. (B) The ability of Vpu S52A to enhance HIV-1 release from Jurkat T-cells is inhibited in a tetherin-dependent manner. 1*10^6 Jurkat cells were electroporated with the different proviral constructs coexpressing GFP and the indicated amount of tetherin expression plasmid as described in the methods section. Two days post electroporation the percentage of GFP+ cells as well as p24 contents of the supernatants were quantified. Presented are means and standard deviations from triplicate electroporations from one representative out of three independent experiments. [file 1742-4690-7-1-S3.PDF]

Figure S3 Schindler et al

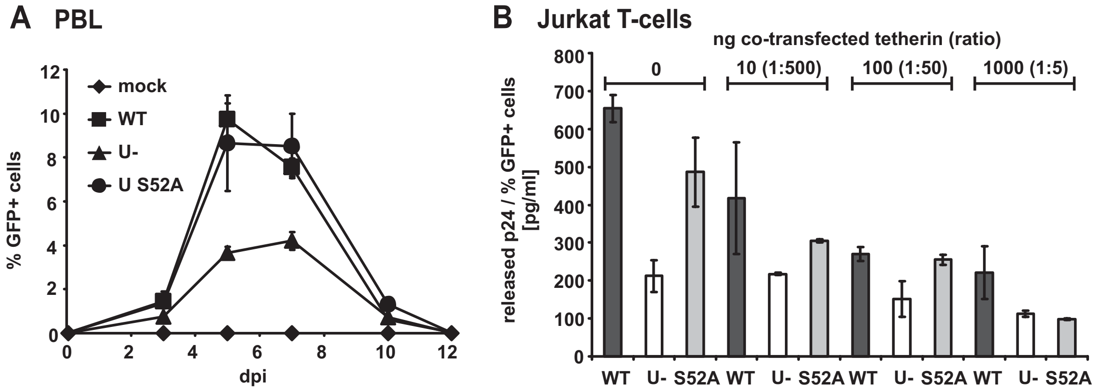

Supplementary Figure S3. Vpu S52A is dispensable for HIV-1 release in primary blood lymphocytes (PBL) and Jurkat T-cells . (**A**) Replication kinetic of the indicated X4-tropic HIV-1 isolates expressing eGFP via an IRES in PBL cultures. PBLs were infected with 1ng p24 and analyzed for the amount of GFP+ cells in two or three days intervals. Means +/- SD are calculated from infections of PBLs from two donors with three independent virus stocks. (**B**) The ability of Vpu S52A to enhance HIV-1 release from Jurkat T-cells is inhibited in a tetherin-dependent manner. 1*10^6 Jurkat cells were electroporated with the different proviral constructs coexpressing GFP and the indicated amount of tetherin expression plasmid as described in the methods section. Two days post electroporation the percentage of GFP+ cells as well as p24 contents of the supernatants were quantified. Presented are means and standard deviations from triplicate electroporations from one representative out of three independent experiments.
